# Nonnegligible Seroprevalence and Predictors of Murine Typhus, Japan (Response)

**DOI:** 10.3201/eid3002.231465

**Published:** 2024-02

**Authors:** Tetsuro Aita, Eiichiro Sando, Shungo Katoh, Sugihiro Hamaguchi, Hiromi Fujita, Noriaki Kurita

**Affiliations:** Fukushima Medical University, Fukushima, Japan (T. Aita, E. Sando, S. Katoh, S. Hamaguchi, N. Kurita);; Kita-Fukushima Medical Center, Fukushima (E. Sando, S. Katoh, H. Fujita)

**Keywords:** murine typhus, Rickettsia typhi, typhus, seroepidemiologic studies, cross-sectional studies, endemic, flea-borne, bacteria, Rickettsia, vector-borne infections, zoonoses, Japan

**In Response:** We thank Dr. Iwata (*1*) for his remarks regarding our study of seroprevalence and predictors of murine typhus in Japan (*2*). In reference to long-term seropositivity, high seroprevalence of *Rickettsia typhi* might reflect distant past murine typhus (MT) infections rather than recent infections (*2*). We acknowledge the significance of this limitation in interpreting our results, which we first addressed in a preprint of the article (T. Aita et al., unpub. data, https://doi.org/10.1101/2023.01.12.23284497). Nonetheless, we posit that *R*. *typhi* seroprevalence would include some persons who have recently experienced MT. First, participants with remarkably high *R. typhi* IgG titers probably had recent MT infections, because *R. typhi* IgG titers generally undergo a continuous postinfection decline in diagnostic serologic assays. The percentage of persons with antibody titers of >1:3,200 in an indirect immunofluorescence assay was ≈85% at 4 weeks postinfection but decreased to ≈25% within 1 year (*3*). In addition, antibody titers continued to decrease over 3 years postinfection in an enzyme-linked immunosorbent assay (*4*). In our study, using an indirect immunoperoxidase-based assay, 20 participants exhibited notably high antibody titers (1:1,280 to >1:40,960). Second, despite raising the diagnostic cutoff from 1:40 to 1:160, the preeminence of *R. typhi* seroprevalence persisted over that of *Orientia tsutsugamushi* (*2*), the causative agent of scrub typhus, which is the most frequent endemic rickettsiosis in Japan. Thus, excluding many persons with distant past infections did not influence the study’s conclusion that *R. typhi* seroprevalence was significantly higher than that of *O. tsutsugamushi*. Therefore, the higher *R. typhi* seroprevalence indicates not only prolonged seropositivity but also recent *R. typhi* infections.

In conclusion, although Dr. Iwata’s commentary is pivotal for a more precise interpretation of our results, our study indicates the occurrence of recent MT cases in Japan. We aim to elucidate this potential MT reemergence by conducting a case-based prospective study.
